# Supplementary material for: Illuminance-tuned collective motion in fish
Source: Commun Biol. 2023 May 31;6:585. doi: 10.1038/s42003-023-04861-8 (PMC10232518; doi:10.1038/s42003-023-04861-8)
Supplement: Supplementary file 2 — Description of Additional Supplementary Files [file 42003_2023_4861_MOESM2_ESM.pdf]

Description of Additional Supplementary Files

File name: Supplementary Movie 1

Description: Collective motion of 53 fish swimming under changing illuminance. Left: Movie recorded at 5 fps with an overhanging camera. Images are recorded in infrared, so that the lighting remains constant when the visible light changes. Top right: Polarization parameter [illegible] _(light blue) and milling parameter [illegible])_ red) with respect to time. The data is averaged over a rolling window of 1 sec. Bottom right: Normalized light intensity $\bar{E}$ with respect to time.
