# Supplementary material for: Illuminance-tuned collective motion in fish
Source: Commun Biol. 2023 May 31;6:585. doi: 10.1038/s42003-023-04861-8 (PMC10232518; doi:10.1038/s42003-023-04861-8)
Supplement: Supplementary file 5 — Illuminance-tuned collective motion in fish [file 42003_2023_4861_MOESM5_ESM.pdf]

Supplementary Material

Illuminance-tuned collective motion in fish

Baptiste Lafoux[1,+], Jeanne Moscatelli[1,+], Ramiro Godoy-Diana[1,+,*], and Benjamin Thiria[1,+,*]

[1]Laboratoire de Physique et Mécanique des Milieux Hétérogènes (PMMH), CNRS UMR 7636, ESPCI Paris—PSL Research University, Sorbonne Université—Université Paris Cité, 10 rue Vauquelin, 75005 Paris, France
[*]ramiro@pmmh.espci.fr, benjamin.thiria@espci.fr

Experiments with fixed light intensity

The primary dataset involves experiments that feature a continuous change in illumination over time. To verify the accuracy of the results and evaluate any potential influence of the duration of the experiment on the behavior of the fish, a new series of experiments was conducted with a consistent light intensity maintained for a prolonged period. In these experiments, approximately 50 fish swam for 60 minutes at a fixed light level, and their motion was recorded a rate of 5 frames per second, using the same experimental setup as for the main experiment.

A total of 8 light levels were selected (with normalized intensities in [0, 0.05, 0.10, 0.15, 0.3, 0.5, 0.75, 1]), and 3 repetitions were performed for each level, in random order. The 200 fish used for these experiments were distinct from those used in the continuous light variation experiments. Two experiments were carried out on each day in the early afternoon, each involving two distinct groups of around 50 fish. Following each pair of experiments, the 100 fish were returned to a separate aquarium, and two additional experiments were conducted with two new groups of fish at least 48 hours later. The two groups were then recombined, and after a week, four further experiments were conducted with four distinct randgroups of 50 fish, according to the same experimental protocol. An example of the timeseries of these experiments is reported on Figure 4 and the results averaged over the replicates in Figure 2.

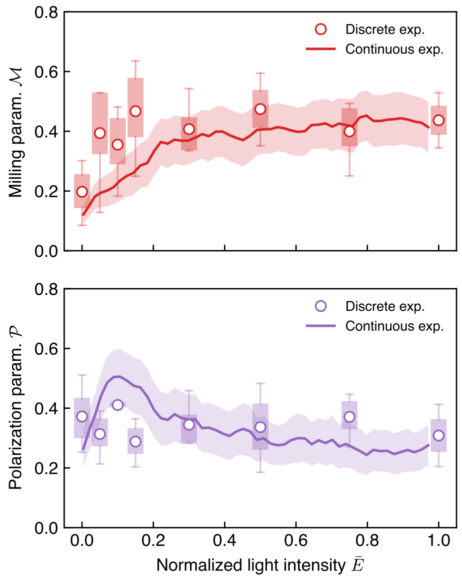

Figure 1. Comparison between discrete (points) and continuous (lines) experiments for schooling parameters (milling and polarization) and distances inside the school (Nearest-Neighbour Distance and Inter-Individual Distance). For continuous experiments, the light intensity is modified gradually over time, while each point for the discrete experiments corresponds to a 1h-experiment at fixed light intensity. The shaded regions around the continuous experiments lines correspond to the 95% confidence interval of the mean. The boxplots show the distribution over the replicates for each light level.

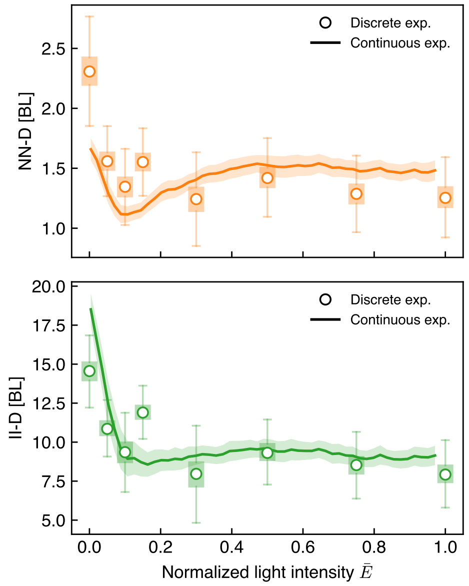

Figure 2. Comparison between discrete (points) and continuous (lines) experiments for distances inside the school (Nearest-Neighbour Distance and Inter-Individual Distance). The shaded regions around the continuous experiments lines correspond to the 95% confidence interval of the mean.

Statistical Analysis

Significance

Friedman test (non-parametric alternative to repeated measurement ANOVA) was conducted to determine whether light intensity had a significant influence on the measured parameters ($\mathscr{M}$, $\mathscr{P}$, NN-D, II-D), over all the different trials for experiments with continous light variations. We binned the data over 8 different intervals of light intensity, with 24 replicates for each of these interval (6 trials, with 4 ramps). The results show a significant influence of the illuminance (see Figure 3) We therefore reject the null hypothesis and conclude that light intensity significantly influences the structure of the school. We then conducted a post-hoc test to the Freidman test, the Nemenyi test, to determine the pairwise comparison of means (the results of the tests are reported in Figure 3).

Role of the duration of the experiment

Complementary 1h-experiments at fixed light intensity have been carried out (see Experiment with fixed light intensity for details). The timeseries of these experiments are reported on Figure 4. Figure 4 shows qualitatively that the behaviour of the fish school is stable over time when no light modification is done. This is true for all measured variables ($\mathscr{M}$, $\mathscr{P}$, NN-D, II-D). In order to determine quantitatively if the time elapsed since the beginning of the experiment have a significant influence on the measurements, we compute Spearman's ρ correlation coefficient for all data. The correlation with time (resp. with light intensity) is computed for experiment with discrete light [24 replicates] (resp. for experiment with continuous light [6 replicates]). Values for the correlation coefficient are reported on Table 1.

For all measured data, we observe a higher correlation with light intensity than with time. This supports the hypothesis that the duration of the experiment has a negligible effect on fish behavior compared to the light intensity, at least for the durations studied here.

Tracking accuracy

The tracking of the fish trajectories is done with the open-source sofware FastTrack[1]. We use the following procedure: we start from the raw images recorded by the camera (.tiff, 2000 x 1000 pixels, about 20 000 images for each experiment). An in-house algorithm is used to extract the background: we select N images randomly in the set of all images (in general N = 10 is largely sufficient). For each pixel, we keep the maximum value among all this subset of images. The fish being darker than the background, this method allows to obtain an image of the tank without fish. This method only works if no place in the tank is occupied in all the images of the selected subset of images, which is largely the case in our experiments since the

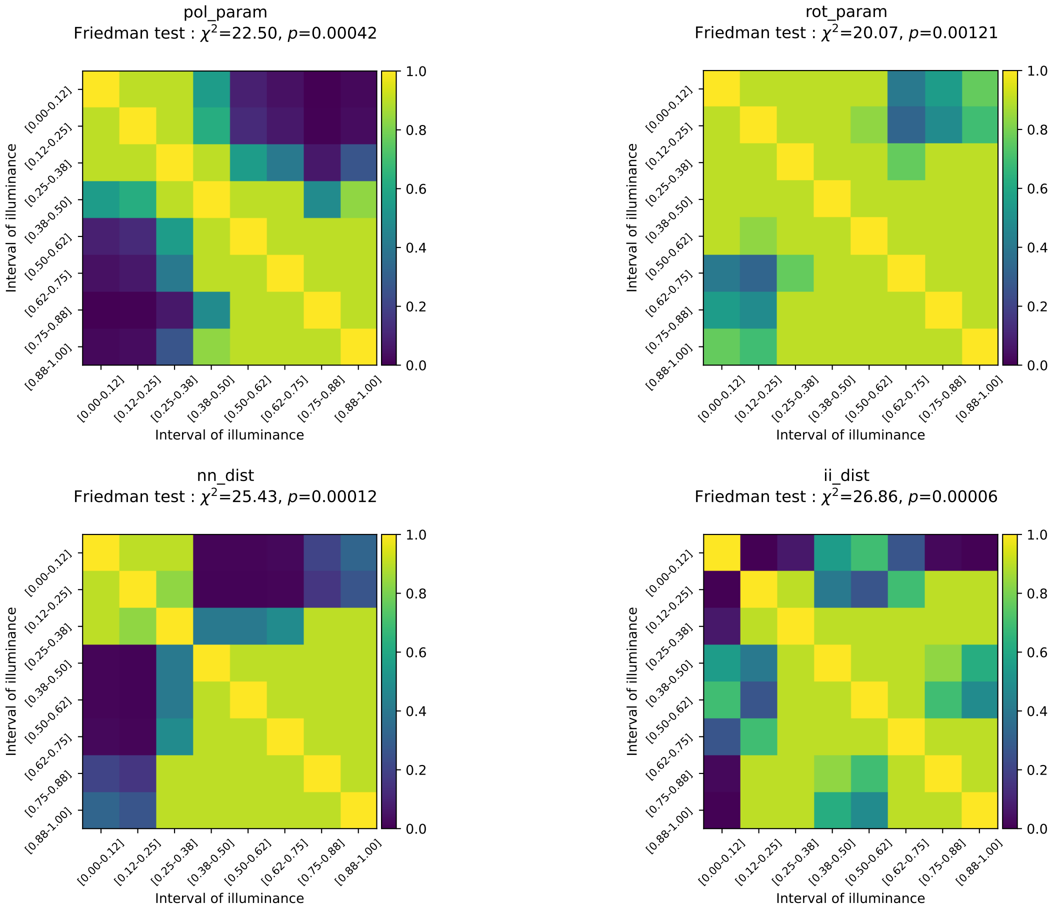

Figure 3. Titles : Results of the Friedman test for each measured variable ($\mathscr{M}$, $\mathscr{P}$, NN-D, II-D). Heatmaps : p-values of the pairwise post-hoc Nemenyi test, that compares that distribution for each light intensity interval two by two.

fish are very mobile. The background obtained in this way is removed from the raw images. The contrast is then improved by normalizing the pixel values in the range 0-1, and a region of interest is selected, slightly larger than the tank size. From the post-processed images, a movie is reconstructed, with the same framerate as the original video recording. The movies in .mp4 format are then used in FastTrack. The tracking parameters chosen are those obtained with the Hungarian algorithm[2] implemented in FastTrack which evaluates them in an optimal way. The head of the animals is tracked.

The binarization of the images is performed by the software. Occlusions (superimposition of two or more fish in the image, when individuals swim above each other for example) are detected when the area of a detected binary blob exceeds a certain threshold (set at 900 pixel). When an occlusion occurs, it is considered that the binary blob concerned does not correspond to any fish. The lost identities are then reassigned when the fish are detected again individually, from the last measured positions/directions. The positions during identity losses are linearly interpolated.

$\mathscr{M}$

Discrete exp. (correlation with time)	[0.002, 0.016, 0.050, 0.023, 0.007, 0.025, 0.000, 0.016, 0.009, 0.030, 0.033, 0.017, 0.031, 0.017, 0.018, 0.028, 0.026, 0.041, 0.010, 0.079, 0.054, 0.004, 0.064]	**avg: 0.0102**
Continuous exp. (correlation with light)	[0.1104, 0.3862, 0.5551, 0.665, 0.3432, 0.1996]	**avg: 0.31005**

$\mathscr{P}$

Discrete exp. (correlation with time)	[0.005, 0.032, 0.025, 0.015, 0.011, 0.026, 0.008, 0.011, 0.026, 0.019, 0.003, 0.001, 0.010, 0.010, 0.006, 0.028, 0.003, 0.042, 0.022, 0.059, 0.117, 0.026, 0.070]	**avg: 0.0055**
Continuous exp. (correlation with light)	[0.2012, 0.6298, 0.4975, 0.6808, 0.1869, 0.2258]	**avg: 0.40366**

II-D

Discrete exp. (correlation with time)	[0.036, 0.024, 0.033, 0.017, 0.071, 0.046, 0.006, 0.021, 0.008, 0.080, 0.025, 0.008, 0.001, 0.000, 0.014, 0.026, 0.006, 0.005, 0.009, 0.021, 0.033, 0.012, 0.014]	**avg: 0.0038**
Continuous exp. (correlation with light)	[0.0366, 0.0342, 0.2916, 0.1223, 0.3494, 0.215]	**avg: 0.16347**

NN-D

Discrete exp. (correlation with time)	[0.034, 0.005, 0.032, 0.036, 0.009, 0.024, 0.001, 0.010, 0.019, 0.120, 0.033, 0.011, 0.002, 0.039, 0.043, 0.016, 0.005, 0.011, 0.001, 0.011, 0.025, 0.002, 0.009]	**avg: 0.0099**
Continuous exp. (correlation with light)	[0.5208, 0.4739, 0.1086, 0.2044, 0.2044, 0.2619]	**avg: 0.29567**

Table 1. Absolute value of Spearman's ρ correlation coefficient

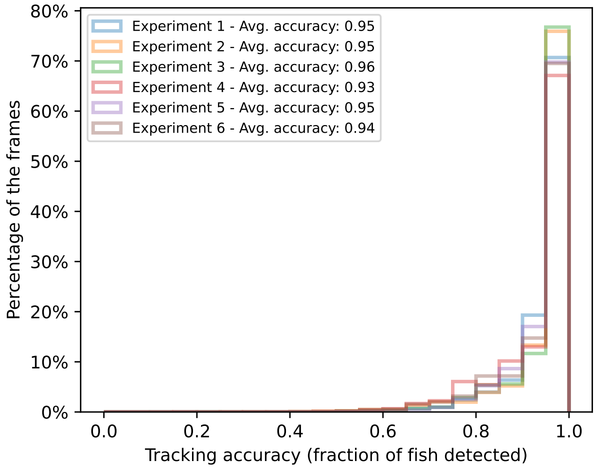

Figure 5. Distribution of the fraction of fish detected on each frame, for each experiment in the main dataset. The average value given in the legend corresponds to percentage of fish detected during the entire experiment.

Figure 5 represents the distribution of the fraction of fish detected per frame. We can see that the majority of fish are detected: on average, for all experiments, more than 90% of the individuals are detected on 86.4% of the frames. Similarly, 94.7% of the fish present in the tank are detected; this represents an average of 1.41 occlusion per frame.

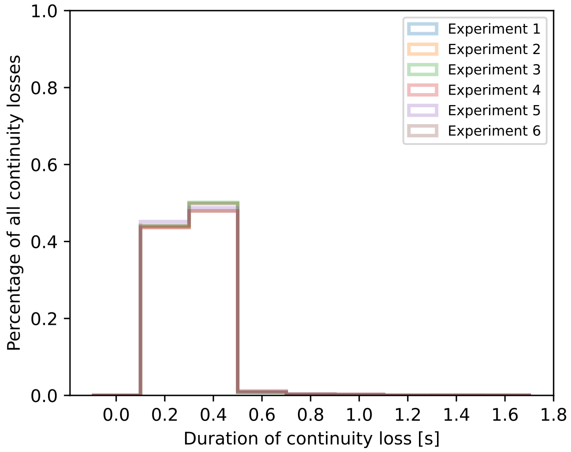

Figure 6. Distribution of the durations of continuity losses (loss of the identities of two or more fish when an occlusion occurs), for all experiments in the main dataset.

Figure 6 represents the distribution of the durations during which the occlusions occur, i.e. the durations during which identities are lost (loss of continuity). We can see that, for all experiments, the majority of the continuity losses (93.2%) have a duration lower than 0.4s (i.e. 2 frames). These data show that the linear interpolation on the positions is performed on short time durations, and suggests that the error made is therefore reasonable.
Overall, these data show that occlusions are an unfrequently occurring situation. Moreover, when occlusions do occur, they are of short duration and the positions can therefore be realistically interpolated. It remains possible that idententies are switched between individual, but this is not a problem for the kind of parameters we measure, since they are global values (computed or averaged at the scale of the entire school).

References

1. Gallois, B. & Candelier, R. FastTrack: An open-source software for tracking varying numbers of deformable objects. *PLoS Comput. Biol.* **17**, 1–19, DOI: 10.1371/JOURNAL.PCBI.1008697 (2021).

2. Kuhn, H. W. The Hungarian method for the assignment problem. *Nav. Res. Logist. Q.* **2**, 83–97, DOI: 10.1002/nav.3800020109 (1955).

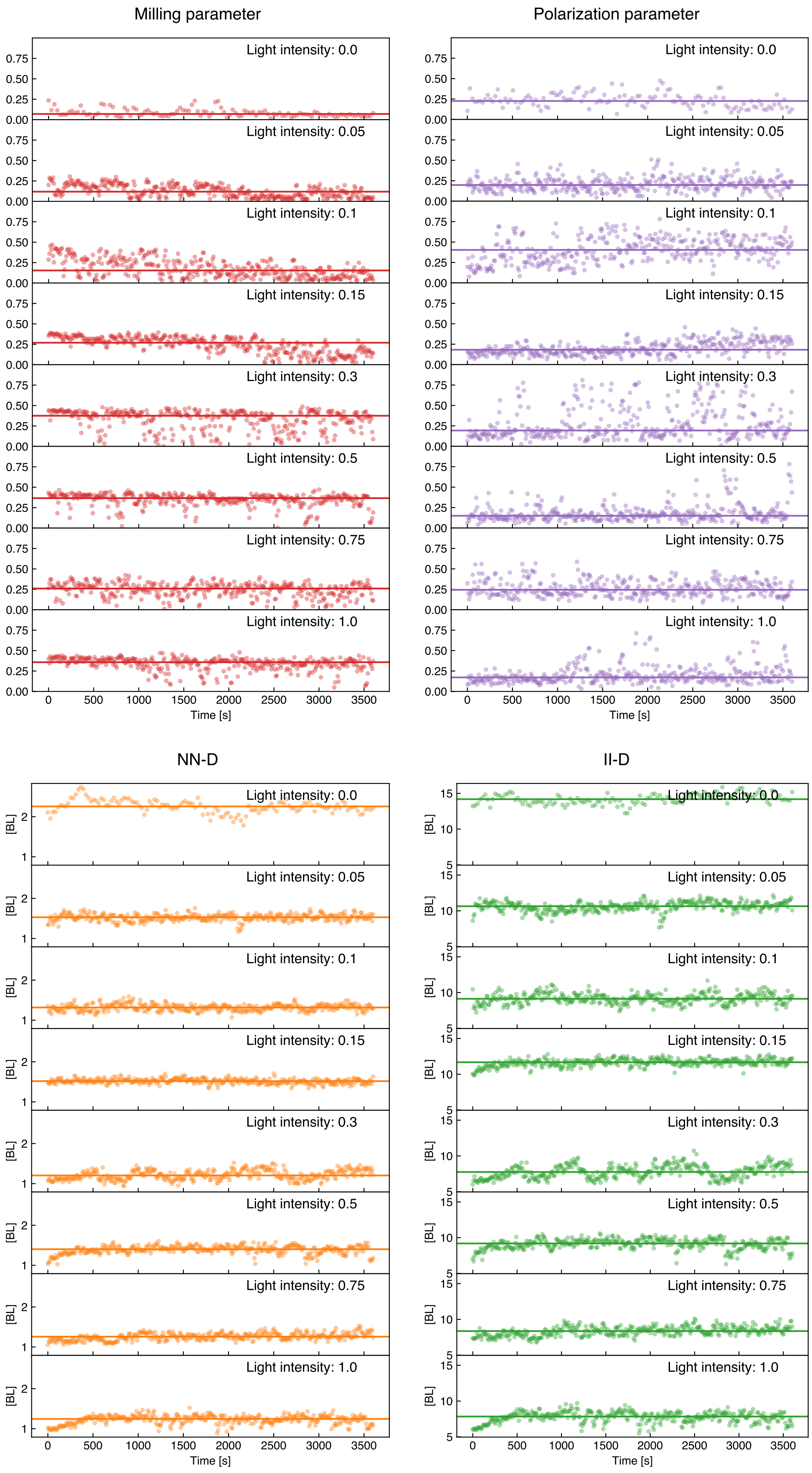

Figure 4. Experimental results with respect to time, for a fixed light intensity. The illumination intensity is fixed during 1 hour. We report here the order parameters and distances in the school. The straight line is the median of the distribution, and points are averaged over 10s.
